# Allergen Immunotherapy: Pitfalls, Perks and Unexpected Allies

**DOI:** 10.3390/ijms26083535

**Published:** 2025-04-09

**Authors:** Tudor Paul Tamaș, Elena Ciurariu

**Affiliations:** 1 Discipline of Immunology and Allergology, Biology, Department of Functional Sciences III, “Victor Babeș” University of Medicine and Pharmacy, Eftimie Murgu Sq. No. 2, 300041 Timişoara, Romania; ciurariu.elena@umft.ro; 2 Discipline of Physiology, Department of Functional Sciences III, “Victor Babeș” University of Medicine and Pharmacy, Eftimie Murgu Sq. No. 2, 300041 Timişoara, Romania; 3 Center of Immuno-Physiology and Biotechnologies (CIFBIOTEH), “Victor Babeș” University of Medicine and Pharmacy, Eftimie Murgu Sq. No. 2, 300041 Timişoara, Romania

**Keywords:** allergen immunotherapy, cellular tolerance, IgG4, biologic therapies

## Abstract

Allergen immunotherapy (AIT) is a well-established treatment aimed at reducing allergen sensitivity by gradually exposing the immune system to increasing doses of allergens. This promotes desensitization and immune tolerance through multiple mechanisms. AIT offers long-term immune modulation and is considered a potentially curative certain forms of allergic diseases. Altered antibody responses is a key mechanism of AIT in the production of allergen-specific IgG4 antibodies, which act as blocking antibodies to prevent allergen binding to IgE on mast cells (MCs) and basophils. However, IgG4 responses are sometimes ineffective due to variations in antibody affinity and epitope targeting. Reverse class switching from IgE to IgG4 and selective depletion of IgE-producing B cells represent potential strategies to improve AIT efficacy. Tregs play a central role in AIT by suppressing Th2-driven allergic responses and promoting immune tolerance through anti-inflammatory cytokines interleukin (IL)-10 and transforming growth factor (TGF)-β. However, genetic and environmental factors may impair Treg function, leading to AIT failure. AIT reduces MC and basophil activation, leading to long-term suppression of allergic inflammation. It modulates IgE-FcεRI interactions and cytokine signaling pathways, but in some cases, anaphylactic reactions or resistance to MC desensitization may occur. Discussion and conclusions: While AIT is a highly effective allergy treatment, variability in immune responses can impact its success. Advances in biologic therapies offer potential synergies with AIT. Understanding these interactions will help refine AIT strategies and improve patient outcomes.

## 1. Introduction

Allergies are a significant public health concern worldwide, with profound implications for patients, healthcare systems, and economies. Allergic diseases affect a substantial portion of the population. In Europe, approximately 30–40% of people suffer from allergic conditions such as rhinitis, asthma, and atopic dermatitis. In the U.S., similar prevalence rates are observed. Allergies can severely impact daily life, causing sleep disturbances, reduced productivity, and impaired mental well-being. For children, allergies can affect school performance and social interactions. Allergies are often underdiagnosed and undertreated, leading to increased morbidity and healthcare utilization [1,2,3].

The direct costs of allergies include medical expenses such as doctor visits, medications, and hospitalizations. In Europe, the annual cost of treating allergic diseases is estimated to be billions of euros. The indirect costs stem from absenteeism (missed workdays) and presenteeism (reduced productivity at work). In the European Union, inadequate management of allergies results in economic losses from EUR 55 to EUR 151 billion annually (2014 estimate). Improved allergy management and early intervention could lead to substantial economic savings. For example, better treatment adherence in Europe could save up to EUR 84 billion annually [4].

Food allergy alone costs the healthcare system in the US approximately EUR 4 billion annually, with an additional EUR 18.9 billion borne by families. There are country-specific disparities, revealing that lower-income groups often incur higher emergency care costs due to limited access to specialized allergy management. Early allergen introduction strategies, such as for peanuts and eggs, have demonstrated potential cost-effectiveness when implemented without prior screening. Still, substantial gaps remain in cost-effectiveness analyses, particularly regarding oral immunotherapy (OIT), which though promising, presents upfront costs and varied outcomes in quality of life and anaphylaxis reduction [5].

Allergen immunotherapy (AIT) is a treatment that has evolved over the past century. It is designed to reduce sensitivity to allergens by gradually exposing the immune system to increasing amounts of the allergen. Several mechanisms are involved in order to reach desensitization, a state where the immune system becomes less reactive to allergens. This is achieved by repeated and controlled exposure to the allergen, presented in native form or allergoids, even as synthetic conjugated proteins more recently, leading to a gradual increase in the threshold for allergic reactions [6,7,8,9].

AIT relies on multiple immune mechanisms which support the following points:

- Immune tolerance: AIT promotes immune tolerance by shifting the overall immune response from a T helper 2 (Th2)-dominated response (which is associated with allergies) to a Th1-dominated response. This shift reduces the production of allergen-specific IgE antibodies and increases the production of tolerogenic antibodies (mainly IgG), which act as blocking antibodies [10,11,12].
- Regulatory T cells (Tregs) induction: AIT stimulates the production of regulatory T cells (Tregs), which play a crucial role in maintaining cellular immune tolerance. Type 1 Treg cells (Tr1) suppress the activity of effector T cells, such as Th2 cells, and reduce inflammation [13,14,15].
- Cytokine modulation: AIT modulates the production of cytokines, which are signaling molecules that regulate immune responses. It increases the production of anti-inflammatory cytokines like Interleukin (IL)-10 and Transforming Growth Factor (TGF)-β, while reducing the production of pro-inflammatory cytokines like IL-4, IL-5, and IL-13 [10,13,16,17,18].
- Effector cell suppression: AIT reduces the activity of effector cells, such as mast cells, basophils, and eosinophils, which are responsible for allergic reactions. This leads to a decrease in the release of histamine and other inflammatory mediators [17,18].

AIT can induce long-term immune tolerance, which may persist even after the treatment is discontinued, after the recommended three years of AIT. This is due to the establishment of a more balanced immune response and the persistence of humoral and cellular immune regulatory mechanisms. These mechanisms work synergically to reduce allergic symptoms and improve the quality of life for individuals with allergies. AIT is considered a potentially curative treatment for allergic diseases, such as allergic rhinitis/conjunctivitis (which are commonly caused by pollen, dust mites, or animal dander), allergic asthma, particularly when triggered by environmental allergens (T2-high endotype), stinging insect hypersensitivity, such as allergies to bee or wasp venom, and in the cases of atopic dermatitis associated with sensitivity to house dust mites or aeroallergens. It is because AIT addresses the underlying immune dysregulation rather than merely alleviating symptoms [19,20,21]. Furthermore, there could be unintended, sometimes unexpected, consequences of immunotherapy in each of its actions listed above, which can prove to be beneficial or detrimental to the patient and will be explored further.

## 2. Altered Antibody Responses

One of the key immune responses induced by AIT is the production of allergen-specific IgG4 antibodies. Recent evidence also highlights a significant and perhaps underestimated role for IgG1, which appears earlier in the course of AIT and demonstrates substantial IgE-blocking activity even before IgG4 levels become prominent. However, IgG1 appears to remain dominant only in a minority of patients (12%) after 12 months of AIT [12].

AIT induces both systemic and local IgA production, which may contribute to immune tolerance by neutralizing allergens, preventing IgE-allergen interactions, and modulating inflammatory signaling via FcαRI on myeloid cells. However, the protective role of IgA remains controversial. Some findings, where mucosal IgA did not differentiate allergic from tolerant individuals to peanut allergens, suggest that high IgA levels do not always correspond with allergen tolerance. These discrepancies may reflect differences in the site and timing of IgA induction as well as isotype-specific effects. Furthermore, while systemic IgA has been linked to inhibition of mast cell degranulation and suppression of IgE-mediated pathways, mucosal secretory IgA’s role remains less clear. The impact of microbiota, cytokine milieu (e.g., TGF-β, IL-21), and glycosylation patterns also complicate interpretations. Thus, although promising, the mechanistic and clinical significance of IgA in AIT remains to be defined, with ongoing questions about the functional contributions of IgA1 versus IgA2 and systemic versus mucosal responses [22].

Some notable points about IgG4 titers induced by allergen immunotherapy include the following points:

- Blocking role: IgG4 antibodies act as blocking antibodies that can prevent allergens from binding to IgE on the surface of mast cells and basophils. This helps reduce the release of histamine and other inflammatory mediators, thereby decreasing allergic symptoms [23].
- Tolerance induction: The production of IgG4 is associated with the induction of immune tolerance. IgG4 can compete with IgE for allergen binding, reducing the overall allergic response. This is particularly important in the context of long-term allergen exposure and repeated dosing during AIT [23,24].
- Clinical efficacy: The presence of allergen-specific IgG4 is considered a biomarker for the clinical efficacy of AIT. Higher levels of IgG4 are often correlated with improved clinical outcomes and reduced allergic symptoms [11,12,25].
- Specific mechanism of action: IgG4 has a unique molecular feature called Fab-arm exchange (FAE), which allows it to bind to two different antigens simultaneously. This property contributes to its ability to neutralize allergens effectively [26,27].
- Non-inflammatory nature: Unlike other IgG subclasses, IgG4 does not activate the complement system and has low affinity for Fc receptors. This makes it an anti-inflammatory antibody, which is beneficial in the context of allergen immunotherapy [24,27,28].

Allergen-specific IgG4 plays a multifaceted role in allergic diseases, acting as a protective factor in some cases and a bystander in others. The induction of high allergen-specific IgG4 titers during AIT is a key component of the therapeutic process, contributing to the overall success of the treatment. However, in each of these instances, IgG responses can be far from perfect. In certain patients, despite reaching meaningful titers, IgG4 antibodies are not blocking (either due to lower affinity or because they are oriented against different epitopes), thus the tolerance induction is delayed or non-existent, hence AIT becomes inefficient [11,12,25,29].

The FAE mechanism can also hinder the ability to neutralize allergens, if the second arm is not involving allergen clearance mechanisms. Natural IgG4 can even be oriented towards autoantigens, thus an increased load of IgG4 due to AIT could potentiate possible autoinflammatory responses (by FAE with autoantigen-specific IgG4). In IgG4-related diseases (IgG4-RD), an increase in autoantibody diversity has also been associated with more severe clinical manifestations. Patients with IgG4-RD typically exhibit heightened IgG4 reactivity to environmental antigens, indicating that elevated IgG4 levels may result from a broad, pleiotropic activation of IgG4+ B cells regardless of antigen specificity. In eosinophilic infiltrate diseases, IgG4 deposits have been diminished during food avoidance tests, but increased in the active phase of the disease, underscoring a potential role at least as an inflammatory marker for these antibodies [30,31,32,33].

During the initial phases of AIT, there may be a transient increase in allergen-specific IgE levels. Over time, with continued treatment, the levels of allergen-specific IgE can decrease. This reduction is associated with the development of immune tolerance and a shift in the immune response from a Th2-dominated response to a Th1-dominated response. Ideally, IgE responses should be dulled during AIT progression; however, in some patients, this is not the case. The IgE/IgG ratio can be a good indicator for AIT efficacy, provided that the affinity of the two antibody types is comparable and that IgG4 exhibits blocking activity [25,34].

Another potential strategy to diminish the humoral immune response is selective B-cell depletion, by means of rituximab (anti-CD20 antibody) or by designing small molecule or peptide-based haptens that specifically bind to membrane IgE; these cells can be selectively recognized and eliminated. The haptens can be conjugated to toxins (such as ribosome-inactivating proteins, apoptosis-inducing drugs, or immunotoxins) or even small-molecule inhibitors that disrupt survival pathways of these B cells [35].

Reverse class switching of IgE-producing B cells towards IgG4, postulated to be triggered in vivo by IL-10 producing regulatory T cells (Tregs) and rendering high affinity IgG4 antibodies, remains an intriguing possibility to be demonstrated in further studies. Reverse class switching is a concept that was hinted at, but not yet achieved in a specific manner. More efficient ways of inducing reverse class switching for specific B cell clones would generate high affinity IgG4 antibodies optimally oriented against relevant IgE epitopes, which would brand such a therapy ideal for achieving humoral tolerance towards allergens [12,24].

## 3. Cellular Tolerance Network

AIT modulates immune response, promoting the expansion of regulatory T cells (Tregs) and production of anti-inflammatory cytokines like IL-10 and TGF-β. These cellular and humoral immune modulators contribute to the suppression of MCs and other effector cells activity, diminishing the allergic inflammatory process. Thus, Tregs play a crucial role in maintaining immune tolerance and preventing allergic reactions. They are involved in multiple processes in AIT. These are as follows:

- Immune tolerance induction: Tregs (specifically Tr1) help induce immune tolerance by suppressing the activity of effector T cells, such as Th2 cells, which are responsible for allergic reactions. This suppression reduces inflammation and allergic symptoms [17].
- Anti-inflammatory cytokine production: Tregs produce anti-inflammatory cytokines like IL-10. Th3 is another population of cells with regulatory roles, which produce mainly TGF-β. These cytokines help modulate the immune response, promoting a more balanced and less reactive state [17,36].
- Suppression of effector cells: Tregs inhibit the activation and function of mast cells, basophils, and eosinophils, which are key players in allergic reactions. This leads to a decrease in the release of histamine and other inflammatory mediators [37].
- Modulation of dendritic cells: Tregs can influence dendritic cells, which are antigen-presenting cells that play a critical role in initiating immune responses. By modulating dendritic cells in a cross-talking manner, Tregs help promote a more tolerogenic environment [38].
- Long-term persistence of immune tolerance: The presence and optimal activity of Tregs are essential for the long-term success of AIT. They contribute to the establishment of sustained immune tolerance, which can persist even after the discontinuation of treatment [39].

There are multiple points of failure where AIT could be rendered ineffective (Table 1). There could be genetic or epigenetic polymorphisms affecting both immune cells populations (functional enhancement for Th2/Th17 and Innate Lymphoid Cell (ILC)2/ILC3, or impairment for Th1/Tr1) and their related molecular machinery (cytokines, receptors, and intracellular signaling) that are involved in the initiation and effector phases of type 2 and type 3 immune responses in allergic inflammation. Alterations in regulatory cytokines (such as IL-12, IL-18, and IL-27), as well as molecules and receptors that control these immune responses and their associated signaling pathways, may also contribute to unsuccessful AIT outcomes. Additionally, AIT failure may result from impaired Th1/Tr1 cell responses, potentially linked to polymorphisms affecting Notch ligand expression and its signaling components. Another critical factor is the disruption of Interferon (IFN)-γ/IFN-γ receptor interactions and related signaling molecules, which may impair the regulatory mechanisms necessary for AIT efficacy. Furthermore, genetic polymorphisms affecting receptors (e.g., histamine receptors, FcεRI) or secreted immunomodulatory molecules (e.g., Indoleamine 2,3-dioxygenase, IDO) could further contribute to AIT failure. Lastly, variations in tolerogenic cytokines (IL-10, IL-35, IL-37, and TGF-β), along with their receptors and signaling pathways, may represent another underlying factor in unsuccessful AIT responses [40,41,42,43,44,45,46,47].

Genetic mutations or polymorphisms in the *FoxP3* gene can lead to dysfunctional Tregs, which may result in autoimmune diseases or reduced immune tolerance. This can affect the efficacy of immunotherapy, as Tregs play a vital role in maintaining immune balance. The FoxP3 protein undergoes several post-translational modifications, such as acetylation, phosphorylation, and ubiquitination. These modifications can influence the stability, localization, and transcriptional activity of FoxP3, thereby potentially affecting Treg function and immunotherapy outcomes. There are also antitumoral therapeutic strategies that target FoxP3, such as antisense oligonucleotides (ASOs), can modulate Treg activity. For example, AZD8701 is a novel ASO that targets the mRNA transcript of *FoxP3* in Tregs, reducing their suppressive function and enhancing anti-tumor immunity, potentially reducing AIT effectiveness [48,49,50].

Prolonged exposure to a chronic inflammatory environment can lead to the loss of FoxP3 protein expression through a mechanism that remains incompletely understood. This process gives rise to a cell population known as exTregs, which are former regulatory T cells (Tregs) that have lost their suppressive function and have transitioned into conventional T effector cells. These exTregs actively contribute to pathogenic immune responses, thereby exacerbating the allergic inflammatory process [51].

The repeated administration of DNA-based vaccines in humans could prompt concerns regarding potential risks. These include the theoretical possibility of plasmid DNA integration into the human genome, the induction of anti-DNA antibodies, and the prolonged existence of allergen stimulation, which may contribute to severe allergic reactions and/or a chronic inflammatory state [52].

Vitamin A and D are known micronutrients that induce Treg population. There can be faster loss of regulatory T cells population with subsequent loss of immune tolerance due to altered response to vitamin D modulation in patients with vitamin D receptor (VDR) polymorphisms [36,53].

## 4. Effector Cell Suppression

AIT is a treatment designed to reduce sensitivity to allergens. The reduced sensitivity translates biologically to induction of tolerance (chiefly serological tolerance by specific IgG induction, as well as cellular tolerance by Treg induction). However, AIT still carries a risk of inducing anaphylaxis, a severe and potentially life-threatening allergic reaction [19].

Mast cells (MCs) play a central role as key effector cells in allergic diseases for several reasons. Strategically positioned as immune sentinels within mucosal and epithelial tissues, they are located in the proximity of the vascular and lymphatic endothelium, allowing them to swiftly detect and respond to allergens. In addition to their advantageous distribution, MCs have a significantly longer lifespan compared to their counterpart effector cell, the basophils. They are capable of retaining surface-bound IgE for extended periods, often persisting for months, and can react to extremely low allergen concentrations. Upon activation, MCs undergo rapid degranulation due to their high content of cytoplasmic granules, which store preformed allergic mediators, enabling an immediate immune response [54,55,56,57,58].

AIT achieves a reduction in mast cell activation, with fewer IgE molecules binding to the high-affinity IgE receptors (FcεRI) on the cell membrane, reducing the MCs activation and degranulation. The AIT mechanisms involved in this reduction in MC IgE binding include a gradual decline in serum allergen-specific IgE levels over time, reducing the overall availability of IgE that can bind to FcεRI, the induction of blocking antibodies, particularly IgG4 and IgA, which compete with IgE for allergen binding, and long-term down-regulation of FcεRI expression on MCs [17,24,39].

AIT inhibits the IgE-mediated release of inflammatory mediators from mast cells, such as histamine, leukotrienes, and cytokines, by the same mechanisms involved in reduction in IgE signaling. This helps reduce the symptoms of allergic reactions, such as itching, swelling, and bronchoconstriction. The JAK-STAT (Janus Kinase–Signal Transducer and Activator of Transcription) pathway is recognized as a key regulatory signaling cascade that operates downstream of cytokine receptors in mast cells and other effector cells. The JAK1/JAK2 inhibitor ruxolitinib was also found to inhibit in vitro IgE-mediated release of preformed (histamine), de novo synthesized mediators (leukotriene C4), and type 2 cytokines (IL-4, IL-13) from human basophils and substance P-mediated release of histamine and other mediators from mast cells. On the other hand, dysregulation of the STAT6 pathway in STAT6-null cells was associated with desensitization failure in MCs [59,60,61].

AIT induces a state of desensitization in mast cells, making them less responsive to allergens. This is achieved through repeated exposure to the allergen, leading to a gradual increase in the threshold for MC activation. The main mechanism in this case appears to be the internalization of IgE-FcεRI complexes [62].

The suppression of mast cells by AIT can lead to long-term benefits, including sustained immune tolerance with a IgE/IgG4 ratio skewed towards the right and reduced allergic symptoms caused by immediate mast-cell mediated reactions, even after the discontinuation of treatment [63].

Studies have shown that IgG4 antibodies can bind to basophils and enhance histamine release when IgE is also present. This indicates that IgG4 can sometimes contribute to allergic reactions rather than blocking them. With regard to the effector cells, the IgE-neutralizing effect of omalizumab, thus far recommended off-label, has been proven convincingly in the case of OIT, venom immunotherapy (VIT), as well as in the case of systemic mastocytosis. In these cases, effector cells are likely hyperactive due to the presence of the allergen-specific IgE and other causes. Furthermore, the anti-IgE agent would lessen the risk of a type I effector response upon AIT [24,64,65,66,67,68,69].

**Figure 1:**
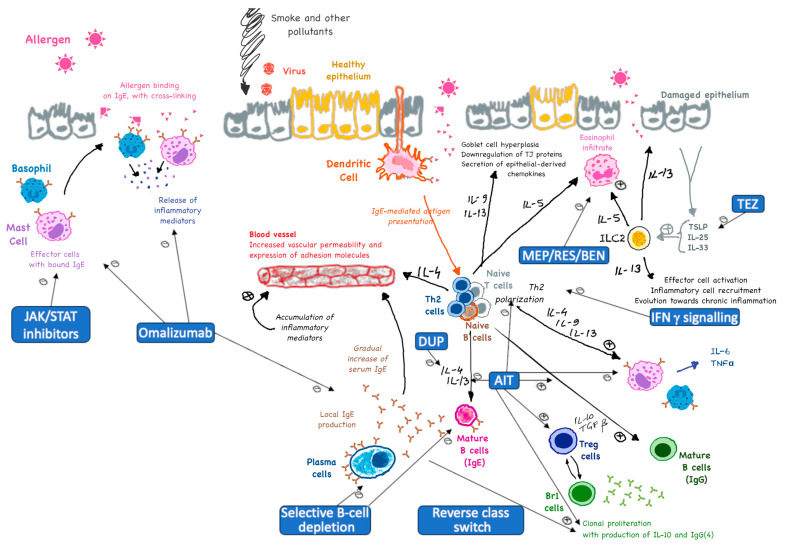
AIT and potential allied therapies—interventions in acute and chronic allergic inflammation. BEN: Benralizumab; Br1: Regulatory B1 cells; DUP: Dupilumab; IFN: Interferon; ILC: Innate Lymphoid Cell, JAK = Janus Kinase; MEP: Mepolizumab; RES: Reslizumab; STAT: Signal Transducer and Activator of Transcription; TEZ: Tezepelumab; TGF: Transforming Growth Factor; TJ: Tight Junction; Treg = Regulatory T cells; TSLP: Thymic Stromal Lymphopoietin).

In addition to omalizumab, several other biologic therapies (Figure 1) have the potential to influence allergen immunotherapy (AIT), especially in the context of runaway Type 2 inflammation or in eosinophilic endotypes of allergic disease. Mepolizumab is a fully humanized IgG1/κ monoclonal antibody that targets interleukin-5 (IL-5) and is used for the treatment of severe eosinophilic asthma. Mepolizumab can reduce eosinophil levels and inflammation, potentially enhancing the effectiveness of AIT. The humanized IgG1/κ monoclonal antibody benralizumab is another IL-5 antagonist that targets the IL-5a receptor subunit on eosinophils and basophils, leading to their depletion. This can help reduce allergic inflammation and possibly improve AIT outcomes. Reslizumab is a humanized IgG4/κ monoclonal antibody. It is similar to mepolizumab in that it targets IL-5 and is also used to treat severe eosinophilic asthma. It can help reduce eosinophil levels and improve the efficacy of AIT. Dupilumab is a monoclonal antibody that targets the IL-4 receptor alpha subunit, inhibiting both IL-4 and IL-13 signaling. Dupilumab is used to treat atopic dermatitis, asthma, and chronic rhinosinusitis with nasal polyps. It can modulate the immune response and enhance the effectiveness of AIT. Preliminary clinical data obtained in studies with limited numbers of patients (*n* = 10 and *n* = 46) indicate a synergistic effect of dupilumab and AIT in patients with moderate to severe AD who are resistant to dupilumab or subcutaneous immunotherapy (SCIT). Tezepelumab is a monoclonal antibody targeting thymic stromal lymphopoietin (TSLP), an epithelial cytokine involved in allergic inflammation. Tezepelumab can reduce type 2 inflammation and improve AIT outcomes. A recent randomized controlled trial revealed that TSLP inhibition by tezepelumab enhances the effectiveness of SCIT during treatment and may facilitate reaching tolerance after only a one-year course of therapy [70,71,72,73,74].

## 5. Discussion and Conclusions

A major immunological outcome of AIT is the induction of allergen-specific IgG4, which acts as a blocking antibody by competing with IgE for allergen binding. This reduces mast cell and basophil degranulation, leading to fewer allergic symptoms. However, IgG4 responses are not always perfectly aligned with tolerance induction. The features of the humoral immune response induced by AIT include blocking of allergen-IgE interactions by IgG4, reducing histamine release, high IgG4 titers that correlate with AIT efficacy, serving as a biomarker and that IgG4 has an anti-inflammatory nature, unlike other IgG subclasses.

Unintended consequences, impacting long-term AIT success, include (1) generation of non-blocking IgG4 leading to inefficient AIT responses in some patients, (2) FAE limitations that could reduce allergen clearance if the second arm fails to engage immune mechanisms, (3) potential induction of IgG4-RD, as, in some cases, elevated IgG4 levels correlate with autoimmune-like inflammatory processes, suggesting a pleiotropic effect of IgG4 on immune tolerance, and (4) IgE persistence in some patients when the expected decrease in allergen-specific IgE does not occur in all individuals.

Unexpected allies in humoral immunity include the concept of reverse class switching with IgE-producing B cells switching to IgG4 via IL-10 modulation. If fully understood and controlled, this could be an ideal mechanism for allergen tolerance. The ratio of IgE to IgG4, rather than absolute levels of either, may be a better predictor of AIT success [75].

Tregs are central to AIT efficacy, mediating immune tolerance by suppressing Th2-driven allergic inflammation. They produce anti-inflammatory cytokines (IL-10, TGF-β) and inhibit effector cells such as mast cells, basophils, and eosinophils. The benefits of the AIT-induced cellular immune response include the suppression of Th2-driven allergic responses by Tregs, promoting immune tolerance, long-term immune reprogramming that allows for sustained remission even after discontinuation of AIT, and the cross-talk between DCs and T cells, which helps shift the immune response toward a tolerogenic profile.

The pitfalls include genetic and epigenetic limitations, which could have been the initial cause of allergy development, such as polymorphisms, in key immune genes that may reduce Treg function, limit effects of regulatory cytokines, and thus impair AIT efficacy. Chronic inflammation can also convert Tregs into pro-inflammatory exTregs, potentially exacerbating allergic reactions. Some cancer therapies interfere with Tregs to enhance immune activation, which may also reduce AIT effectiveness. The theoretical risk of DNA integration or chronic allergen stimulation remains a consideration in future AIT advancements. Variability in VDR function could impact Treg induction and tolerance maintenance.

Unexpected allies are represented by (1) a possible tolerogenic spillover effect, when AIT may reduce or delay autoimmune disease onset by expanding Treg populations, a phenomenon that could have implications for autoimmune disease prevention, and (2) some drugs that disrupt Treg function in cancer, while others could potentially enhance Treg stability for AIT applications.

AIT reduces mast cell and basophil activation, leading to long-term suppression of allergic inflammation. The IgE-FcεRI interaction is gradually diminished, decreasing the potential for anaphylactic reactions. Advantages of AIT-driven effector cell modulation include a diminished mast cell activation leading to reduced histamine release, downregulation of FcεRI expression on mast cells that reduces allergen sensitivity, and long-term desensitization leading to sustained tolerance after AIT discontinuation.

The hidden risks include (1) the possibility of anaphylaxis as, despite tolerance induction, AIT still carries a risk of severe allergic reactions, particularly in patients with high baseline IgE levels, (2) the IgG4 paradox, that is, when IgG4 generally reduces mast cell degranulation, it can enhance histamine release under certain conditions when IgE is also present, (3) failure of mast cell desensitization (e.g., if STAT6 signaling is disrupted, then mast cells may remain hyperactive), reducing AIT efficacy, and (4) JAK-STAT dysregulation, as while JAK inhibitors (e.g., ruxolitinib) can block IgE-mediated signaling, their effects on long-term AIT remain uncertain.

The largest drawback of the AIT routes (subcutaneous, sublingual, and oral) currently in use (Table 2) is the systemic exposure and direct contact of the effector cells with the allergen in previously sensitized individuals. Alternative administration routes include epicutaneous and intralymphatic immunotherapy (EPIT, ILIT). EPIT involves the application of allergens to intact skin via a patch, stimulating Langerhans cells and dermal dendritic cells without systemic exposure. EPIT has shown promise, particularly in pediatric food allergy, due to its favorable safety profile; however, its efficacy is modest compared to oral routes, likely due to the limited penetration of the allergen through the skin barrier. Therefore, EPIT may require a longer time to achieve sustained tolerance. Another emerging route is ILIT, which delivers small doses of allergens directly into a lymph node under ultrasound guidance. ILIT offers the advantage of requiring only a few injections to achieve clinical benefits, significantly reducing treatment duration. Initial studies report good tolerability and encouraging efficacy, especially in allergic rhinitis. However, the invasiveness of this procedure may hinder long-term adherence, even though it may require less frequent administration, as the allergen is placed in situ with the tolerogenic cells (B cells, naïve T cells, and Th1 and Tr1 cells) and it bypasses the peripheral cells, which may be impaired in their tolerogenic response due to various factors (such as altered microbiota, pollution, and barrier defects) [18,76,77,78,79].

Biological therapies with the potential to enhance AIT results are the unexpected allies acting mainly on the effector cells. Firstly, omalizumab (anti-IgE therapy) reduces free IgE levels, lowering FcεRI expression and improving AIT safety. This agent has already proven effective in food allergy desensitization, venom immunotherapy, and systemic mastocytosis. It is likely that omalizumab will also prove its usefulness in other instances of AIT-related anaphylaxis. Mepolizumab, benralizumab, and reslizumab (anti-IL-5 therapies) reduce eosinophilic inflammation, potentially improving AIT outcomes in eosinophilic endotypes. Regarding dupilumab (anti-IL-4/IL-13 therapy), preliminary data suggest synergistic effects when combined with AIT in moderate-to-severe atopic dermatitis; this biological therapy could be employed in other pathologies in the future. Tezepelumab (anti-TSLP therapy) facilitates tolerance induction and may allow faster AIT desensitization [64,65,66,67,68,69,70,71,72,73,74].

In conclusion, AIT is a powerful and effective tool for allergen desensitization, yet it presents multiple challenges and unpredictable outcomes in some patients. The interplay between antibody responses, cellular tolerance mechanisms, and effector cell suppression will ultimately determine the overall success of AIT.

## Figures and Tables

**Table 1:** Mechanisms potentially influencing allergic inflammation and AIT effectiveness.

Altered Component	Mechanism	Potential Influence on Allergy/AIT
STAT3	↑ STAT3 → Th17 differentiation and ILC3 cytokine production (IL-17, IL-22)	Exacerbated Th17/ILC3-mediated inflammation, particularly in mucosal tissues
STAT5	↑ STAT5 activation via IL-2 → ILC2 expansion and can suppress Treg stability	Amplified type 2 immunity via ILC2 expansion, impaired regulation via unstable Tregs
STAT6	↑ STAT6 via IL-4/IL-13 → enhanced Th2 polarization and ILC2 activation	Heightened Th2/ILC2 responses in asthma, atopic dermatitis, eosinophilia
STAT1	↓ STAT1 via impaired IFN-γ signaling, weakened Tr1 tolerance induction and Th1 response	Loss of Th1-mediated counterbalance to Th2 with enhanced allergic inflammation
STAT4	↓ STAT4 → deficient IL-12-mediated Th1 polarization and IFN-γ production	Failure to suppress Th2 expansion with increased allergic sensitivity
Notch Ligands (e.g., Jagged1, Delta-like 4)	↑ Jagged1 on dendritic cells promotes Th2/ILC2 polarization via Notch2 → enhanced type 2 immunity	Bias toward Th2 differentiation with enhanced allergic sensitization and airway inflammation
IFN-γ/IFN-γ Receptor Signaling	↓ IFN-γR expression or STAT1 signaling → impaired Th1 differentiation, reduced antagonism of Th2/Th17 responses	Loss of immune counter-regulation with unchecked Th2/Th17-driven allergic inflammation
GATA3, IL-4 signaling	↑ Th2 signaling, resistance to Treg suppression	Enhanced eosinophilia, IgE-skewed class switching, asthma
RORγt, IL-23	↑ Th17 RORγt, IL-23 responsiveness, GM-CSF co-expression	Neutrophilic inflammation, steroid-resistant airway disease
IL-33/IL-25	↑ ILC2 IL-33/IL-25 sensitivity, loss of PD-1/IL-10R regulation	Exaggerated airway reactivity, fibrosis, atopic dermatitis
IL-23R	↑ ILC3 IL-23R signaling, plasticity toward IL-17+ ILC3s	Chronic intestinal inflammation, epithelial hyperreactivity
T-bet, IL-12	↓ Th1 T-bet or IL-12 signaling, impaired IFN-γ response	Failure to counterbalance Th2 response with worsened allergic inflammation
IL-10	↓ Tr1 IL-10 production → failure to suppress Th2/Th17 responses	Insufficient control of effector T cells with sustained allergic responses
IL-18	↑ IL-18 in allergic tissues → Th2/ILC2 activation in synergy with IL-33	Amplification of type 2 inflammation, eosinophilia, and airway hyperreactivity
IL-27	↓ IL-27 → diminished inhibition of Th2/Th17 and reduced IL-10 induction	Loss of immune regulation with sustained allergic inflammation, reduced tolerance

STAT: Signal transducer and activator of transcription; ROR: Retinoic acid-related orphan receptor; GATA: GATA-binding protein; GM-CSF: Granulocyte–macrophage colony-stimulating factor; PD-1: Programmed cell death protein 1, ↑: increased, ↓: decreased, →: results in.

**Table 2:** Recent clinical trials investigating allergen and allergoid immunotherapy and associated biologics (source: EudraCT, https://euclinicaltrials.eu/search-for-clinical-trials/?lang=en, accessed on 27 March 2025).

Trial ID	Title	Status
2023-508013-16-00	A prospective, randomized, double-blind placebo-controlled multicenter study with mannan-conjugated birch pollen allergoids administered subcutaneously to adolescent and adult patients with birch pollen-induced allergic rhinitis or rhinoconjunctivitis.	Ended
2022-502366-25-00	A three-year, multi-center, double-blind, extension study to evaluate the long-term safety and efficacy of ligelizumab in patients who completed ligelizumab’s Phase III studies in food allergy	Ended
2022-502984-39-00	A multicenter, randomized, double-blind, parallel-group placebo-controlled, Phase III, efficacy and safety study of tezepelumab in 5-to-<12-year old children with severe uncontrolled asthma (HORIZON)	Ongoing, recruiting
2024-511383-88-00	A Phase II–III study to assess the efficacy and safety of subcutaneous cluster-immunotherapy in patients suffering from Olea europaea pollen allergy	Ongoing, recruitment ended
2023-505567-37-00	A Phase II–III study to assess the efficacy and safety of sublingual immunotherapy in patients suffering from birch pollen allergy	Ended
2023-505880-35-00	A Phase II–III study to assess efficacy and safety of sublingual immunotherapy in patients suffering from grass pollen allergy	Ended
2023-504942-75-01	A randomized, double-blind, placebo-controlled, multi-center study to assess the efficacy of PURETHAL Mites mixture 50,000 AUeq/mL subcutaneous immunotherapy in adult subjects with moderate to severe allergic rhinitis/rhinoconjunctivitis with or without asthma induced by house dust mite (HDM) allergy	Ongoing, recruitment ended
2022-502110-85-00	A Phase III, double-blind, placebo-controlled, randomized study to assess the efficacy and safety of epicutaneous immunotherapy with DBV712 250 μg in 4–7-year-old children with peanut allergy (VITESSE)	Ongoing, recruitment ended
2022-503053-19-00	A clinical study investigating OM-85-IN safety and tolerability in healthy volunteers and mild allergic asthma patients	Ended
2024-515717-17-00	A prospective, randomized, double-blind placebo-controlled multicenter trial with mannan-conjugated birch pollen allergoids administered subcutaneously to adolescents and adults with birch pollen-induced allergic rhinitis or rhinoconjunctivitis.	Ongoing, recruitment ended
2023-508520-36-00	A long-term, double-blind, randomized, placebo-controlled clinical trial to investigate the efficacy and safety of PQ Grass 27,600 SU in children and adolescents with seasonal allergic rhinitis/rhinoconjunctivitis induced by grass pollen exposure	Ongoing, recruiting
2023-508817-18-00	A Phase III, multicenter, randomized, double-blind, placebo-controlled, parallel group study to evaluate the efficacy and safety of lebrikizumab/LY3650150 in adult participants with perennial allergic rhinitis	authorized, recruiting
2023-509833-38-00	A multi-national Phase IIIb, double-blind, placebo-controlled trial to determine the safety and efficacy of STALORAL^®^ Birch 300 IR in children and ddolescents of 5 to 17 years old with birch pollen-induced allergic rhinoconjunctivitis with or without asthma	Ongoing, recruitment ended

## Data Availability

All the data are contained within the article.
